# The Interplay Between High Cumulative Doses of Radioactive Iodine and Type 2 Diabetes Mellitus: A Complex Cardiovascular Challenge

**DOI:** 10.3390/ijms26010037

**Published:** 2024-12-24

**Authors:** Adina Elena Stanciu, Madalina Lucica Bolovan, Adina Zamfir-Chiru-Anton, Catalina Voiosu, Pradeep Kumar Dabla, Marcel Marian Stanciu, Nafija Serdarevic, Mirela Gherghe

**Affiliations:** 1 Carcinogenesis and Molecular Biology Department, Institute of Oncology Bucharest, 022328 Bucharest, Romania; madalina.bolovan@iob.ro; 2 Faculty of Biology, University of Bucharest, 050095 Bucharest, Romania; 3 ENT Department, “Grigore Alexandrescu” Children’s Emergency Hospital, 011743 Bucharest, Romania; zamfiradina@yahoo.com; 4 ENT Department, University of Medicine and Pharmacy “Carol Davila” Bucharest, 050474 Bucharest, Romania; catalina.pietrosanu@umfcd.ro; 5 ENT Department, “Prof. Dr. Dorin Hociota” Institute of Phonoaudiology and Functional ENT Surgery, 050751 Bucharest, Romania; 6 G.B. Pant Institute of Postgraduate Medical Education & Research (GIPMER), Delhi 110002, India; pradeep_dabla@yahoo.com; 7 Electrical Engineering Faculty, University “Politehnica” of Bucharest, 060042 Bucharest, Romania; marcel.stanciu@upb.ro; 8 Institute for Clinical Chemistry and Biochemistry, University of Sarajevo Clinics Center, 7100 Sarajevo, Bosnia and Herzegovina; serdarevicnafija@yahoo.com; 9 Nuclear Medicine Department, University of Medicine and Pharmacy “Carol Davila” Bucharest, 050474 Bucharest, Romania; mirela.gherghe@umfcd.ro; 10 Nuclear Medicine Department, Institute of Oncology Bucharest, 022328 Bucharest, Romania

**Keywords:** differentiated thyroid cancer, type 2 diabetes mellitus, radioiodine, cardiotoxicity, metformin, cardiac biomarkers

## Abstract

Starting from the metabolic profile of type 2 diabetes mellitus (T2DM), we hypothesized that the mechanisms of ¹³¹I-induced cardiotoxicity differ between patients diagnosed with differentiated thyroid cancer (DTC) with/without T2DM, with metformin potentially acting as a cardioprotective agent by mitigating inflammation in patients with T2DM. To address this hypothesis, we quantified, using ELISA, the serum concentration of several key biomarkers that reflect cardiac injury (NT-proBNP, NT-proANP, ST2/IL-33R, and cTn I) in 74 female patients with DTC/−T2DM and 25 with DTC/+T2DM treated with metformin. All patients received a cumulative oral dose of ^131^I exceeding 150 mCi (5.55 GBq) over approximately 53 months. Our results showed the following: (i) In DTC/−T2DM patients, high-cumulative ^131^I doses promote a pro-inflammatory state that accelerates the development of cardiotoxicity. Monitoring NT-proBNP, ST2/IL-33R, and cTn I in these patients may help identify those at risk of developing cardiac complications. (ii) In patients with DTC/+T2DM, high-cumulative ^131^I doses lead to the release of NT-proANP (r = 0.63), which signals that the atria are under significant stress. (iii) In patients with DTC/+T2DM, metformin suppresses inflammation, leading to a dose-dependent reduction in cTn I (r = −0.59). Monitoring cTn I and NT-proANP, and considering the use of metformin as part of the therapeutic strategy, could help manage cardiotoxicity in T2DM patients undergoing ^131^I therapy.

## 1. Introduction

In the 10th Edition of IDF Diabetes Atlas, the prevalence of diabetes is estimated to increase from 537 million in 2021 to around 783 million by 2045 [1], highlighting the urgent need for effective strategies to manage and prevent this increasingly challenging disease worldwide. Type 2 diabetes mellitus (T2DM) is a chronic metabolic disorder characterized by disruptions in glucose metabolism [2], and it accounts for approximately 98 percent of diabetes cases worldwide [3]. The relationship between T2DM and differentiated thyroid cancer (DTC) is an open source for research. The existing evidence suggests that individuals with DTC who undergo a total thyroidectomy may be at an increased risk of developing T2DM, with this risk escalating to 40%, regardless of their age [4]. The potential mechanisms connecting T2DM and DTC include several factors, such as hormonal imbalances, elevated insulin levels (hyperinsulinemia), and chronic inflammation [5]. In addition, the complete suppression of the thyroid-stimulating hormone (TSH) after a total thyroidectomy, achieved using a high dose of levothyroxine, might negatively impact glucose homeostasis in individuals with DTC [6]. The interaction of these factors highlights the strong crosslinks between T2DM and DTC.

Radioactive iodine (^131^I) therapy is a standard approach, following surgery, for treating DTC patients without T2DM (DTC/−T2DM) and those with T2DM (DTC/+T2DM) [7,8]. However, this treatment may have some adverse effects on the cardiovascular (CVD) system (heart and blood vessels), encompassing the following: (i) increased CVD mortality in patients who received high cumulative doses of ^131^I [9]; (ii) elevated incidence of subsequent malignancies in various organs, including the heart [10]; and (iii) augmented risk of developing atrial fibrillation, hypertension, coronary artery disease, and heart failure [11]. These risks depend on variables such as the high cumulative ^131^I dose, therapy frequency, the patient’s age and gender, other medical conditions, including T2DM, and the duration of post-treatment follow-up. The focus on the effect of a high cumulative ^131^I dose on DTC/+T2DM patients is driven by several critical scientific and clinical rationales as follows: the impact of T2DM on radiation sensitivity (altered cellular metabolism due to insulin resistance and chronic low-grade inflammation [5]; altered pharmacokinetics or biodistribution of ^131^I due to T2DM metabolic condition [2]; the impairment of renal function [2] which may increase the risk of radiation exposure to non-target tissues and organs) and potential synergistic or antagonistic effects (diabetes-associated complications exacerbation, such as cardiovascular dysfunction [2] or impaired ^131^I efficacy or toxicity due to diabetes-related pathways, requiring dose adjustments or modified treatment protocols). Understanding the interplay between a high cumulative ^131^I dose and T2DM would optimize targeted therapy by adapting treatment strategies, improving therapeutic outcomes, minimizing side effects, and developing adjuvant therapies to mitigate the adverse effects.

Metformin (1,1-dimethylbiguanide hydrochloride), as a monotherapy or combined with sulfonylureas or dipeptidyl peptidase-4 inhibitors, is the first-line medication of T2DM in most guidelines [12]. Even though almost 100 years have passed since its synthesis in 1922, the mechanisms underlying its therapeutic action still need to be fully understood. For example, although the data suggest that metformin (an FDA-approved antidiabetic agent) administration inhibits iodine uptake by thyroid cells and thus may limit the effectiveness of ^131^I treatment [13], metformin may also target thyroid cancer growth through cellular metabolism [14,15,16,17]. Metformin use during breast radiotherapy was associated with the reduced radiation-induced cardiac toxicity in women with early-stage breast cancer [18]. Moreover, recent results showed the radioprotective effect of metformin explained by its indirect modulation of the gene expression involved in cellular detoxification in mice injected with metformin for three days before exposing them to whole-body radiation or a simulation of galactic cosmic rays, which occurs during space travel, at the NASA Space Radiation Laboratory [19]. Surprisingly, metformin, a first-choice drug for glucose lowering in T2DM patients, could have broad implications for cancer patients, nuclear accident response teams, and even astronauts traveling in deep space. However, its potential to modulate the effects of ^131^I therapy as a radiosensitizer for cancer cells, while protecting healthy tissues, is underexplored. Moreover, the risks of ^131^I therapy in the T2DM population are unexplored: compounding risks and bystander effects (there is little evidence on how a diabetes-induced systemic inflammation may influence non-target tissue damage). More research is needed on metformin as a modifier of ^131^I outcomes and on addressing possible gaps in clinical practice guidelines. Unfortunately, the current guidelines for ^131^I therapy [20] do not adequately address the risks in diabetic patients, leading to uncertainty regarding optimal dosing, safety monitoring, and the use of adjuvant therapy.

The recent guidelines on cardio-oncology developed in collaboration with the European Hematology Association, the European Society for Therapeutic Radiology and Oncology and the International Cardio-Oncology Society [21] have emphasized the significance of cardiac-specific biomarkers, including B-type natriuretic peptide (BNP) and cardiac troponin (cTn), in enhancing the traditional echocardiographic assessment of cardiotoxicity linked to cancer treatments. Because there is a need for a greater consensus on the biomarkers that should be used to monitor radiation-induced cardiotoxicity in patients undergoing ^131^I therapy, we selected several biomarkers whose role in radiation-induced cardiac damage, in the presence of T2DM and metformin treatment, should be better defined. N-terminal proBNP (NT-proBNP), the inactive precursor of BNP, and N-terminal pro atrial natriuretic peptide (NT-proANP), the inactive precursor of ANP, are enzymatically cleaved to produce an active peptide that regulates blood pressure and fluid balance. The increased levels of NT-proBNP in patients undergoing cancer therapy reflect the extent of cardiac dysfunction and remodeling [21,22]. Elevated cardiac troponin I (cTn I) levels in patients experiencing cancer therapy-induced cardiotoxicity indicate the degree of myocardial necrosis [21,22,23]. Additionally, several non-cardiac-specific biomarkers have emerged, shedding light on crucial pathophysiological features of radiation-induced cardiotoxicity, such as inflammation, oxidative stress, extracellular matrix remodeling, neurohormonal activation, and myocyte injury [24]. Among these biomarkers, ST2 [also known as receptor of interleukin-33 (IL-33R)], the product of the gene IL1RL1 (interleukin-1 receptor-like 1; GeneID:9173), stands out due to its demonstrated efficacy as an additional stratification factor for heart failure patients, revealing cardiac stress and fibrosis [25]. Its role in chemo/radiation-induced cardiotoxicity may involve its ability to reflect inflammatory and fibrotic processes occurring in response to cancer therapy [26,27].

Considering the above, the study will attempt to answer the following questions:

(i) What are the mechanisms by which high cumulative doses of ^131^I cause cardiotoxicity in patients with DTC with/without T2DM?
(ii) Can the anti-inflammatory effects of metformin reduce the level of radiation-induced myocardial injury in patients with T2DM?
(iii) Can specific biomarkers, such as NT-proBNP, NT-proANP, ST2/IL-33R, and cTnI, be considered as predictive markers of high cumulative ^131^I dose-specific induced cardiotoxicity?
(iv) Does radiation exposure in T2DM patients lead to long-term complications, like diabetic cardiomyopathy or heart failure, and can metformin prevent or delay these outcomes?

## 2. Results

### 2.1. Characteristics of the Study Population

Table 1 presents some of the clinical, hematological, and biochemical parameters that provide insights into the difference between the two groups of patients (DTC/−T2DM group vs. DTC/+T2DM group). The multivariable analysis showed that age was not a confounding factor and that there was no significant difference in age between the two groups. However, the difference in body mass index (BMI) between the two groups was statistically significant (*p* = 0.003) (Table 1), underscoring its importance. Patients with DTC/−T2DM were classified as overweight with a median BMI of 29 kg/m^2^, whereas those with DTC/+T2DM were classified as obese class I with a median BMI of 34.6 kg/m^2^ [28]. Moreover, the multivariable analysis showed that BMI was a confounding factor in the DTC/+T2DM group. Regarding levothyroxine treatment, the two groups had no significant difference in the daily dose and median TSH level.

Furthermore, significant differences in the absolute number of lymphocytes and platelets were observed in Table 1, with the DTC/+T2DM group showing an increased platelet count (*p* = 0.002). The study also analyzed a multi-biomarker panel and found that NT-proBNP, cTn I, and ST2/IL-33R showed statistically significant differences between the two groups (*p* < 0.05). NT-proBNP and ST2/IL-33R serum concentrations were higher (*p* = 0.001 and *p* = 0.033, respectively), and cTn I was lower (*p* = 0.013), in patients with DTC/+T2DM than those with DTC/−T2DM. To summarize, our study found that patients with DTC/+T2DM received a higher cumulative dose of ^131^I, had higher circulating levels of NT-proBNP and ST2/IL-33R, lower circulating levels of cTn I, and showed differences in the absolute number of lymphocytes and platelets, compared to patients without T2DM, providing a comprehensive understanding of the differences between the two groups.

### 2.2. Correlations in the DTC/−T2DM Group

The correlations presented in the DTC/−T2DM group suggest that cumulative doses of ^131^I promote a pro-inflammatory state that accelerates the development of cardiotoxicity.

The cumulative dose of ^131^I triggers inflammation, as reflected in the negative correlation with the absolute lymphocyte count (r = −0.58, *p* < 0.001) and positive correlation with the neutrophil-to-lymphocyte ratio (NLR) and the platelet-to-lymphocyte ratio (PLR) (r = 0.57 and r = 0.46, *p* < 0.001).

**Figure 1:**
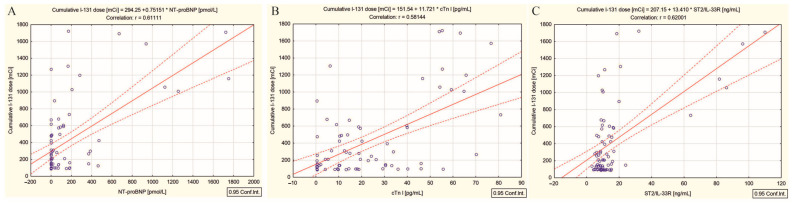
Correlations between (**A**) the cumulative doses of ^131^I and NT-proBNP, (**B**) the cumulative doses of ^131^I and cTn I and (**C**) the cumulative doses of ^131^I and ST2/IL-33R in differentiated thyroid cancer patients without type 2 diabetes mellitus; (“—” fitted linear regression curve, “- - -” equality line).

As for the panel of analyzed cardiac biomarkers, Figure 1 scatter plots demonstrate a positive relationship between cumulative ^131^I doses and NT-proBNP (r = 0.61, *p* < 0.001) (Figure 1A), cumulative ^131^I doses and cTn I (r = 0.58, *p* < 0.001) (Figure 1B), and cumulative ^131^I doses and ST2/IL-33R (r = 0.62, *p* < 0.001) (Figure 1C). However, NT-proANP was not correlated with the cumulative doses of ^131^I.

**Figure 2:**
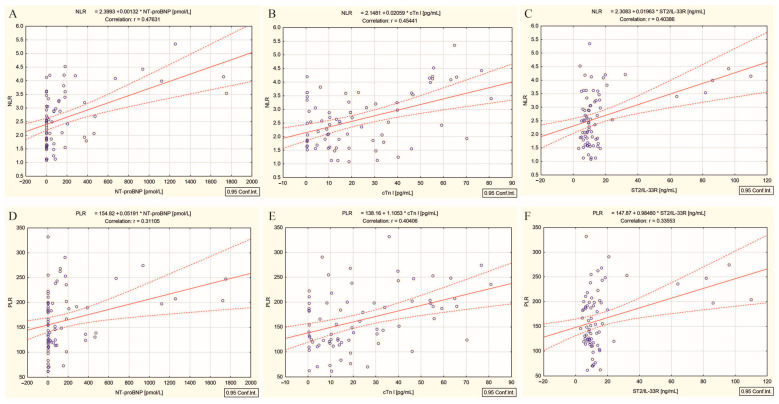
Correlations between (**A**) NLR and NT-proBNP, (**B**) NLR and cTn I, (**C**) NLR and ST2/IL-33R, and between (**D**) PLR and NT-proBNP, (**E**) PLR and cTn I, (**F**) PLR and ST2/IL-33R in differentiated thyroid cancer patients without type 2 diabetes mellitus; (“—” fitted linear regression curve, “- - -” equality line).

The high-dose radiation-induced inflammatory response biomarkers, NLR and PLR, were positively correlated with NT-proBNP (r = 0.47, *p* < 0.001 and r = 0.31, *p* = 0.007) (Figure 2A,D), cTn I (r = 0.45, *p* < 0.001 and r = 0.40, *p* < 0.001) (Figure 2B,E), and ST2/IL-33R (r = 0.40, *p* < 0.001 and r = 0.33, *p* = 0.003) (Figure 2C,F).

**Figure 3:**
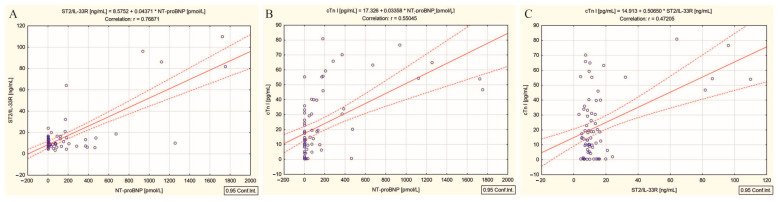
Correlations between (**A**) ST2/IL-33 and NT-proBNP, (**B**) cTn I and NT-proBNP, and (**C**) cTn I and ST2/IL-33R in differentiated thyroid cancer patients without type 2 diabetes mellitus; (“—” fitted linear regression curve, “- - -” equality line).

Moreover, in response to radiation injury, the three biomarkers previously mentioned correlated with each other as follows: ST2/IL-33R with NT-proBNP (r = 0.76, *p* < 0.001) (Figure 3A), NT-proBNP with cTn I (r = 0.55, *p* < 0.001) (Figure 3B), and ST2/IL-33R with cTn I (r = 0.47, *p* < 0.001) (Figure 3C).

The lack of a correlation between NT-proANP, a hormone involved in regulating blood pressure and fluid balance, and the cumulative doses of ^131^I, NLR, PLR, NT-proBNP, cTn I, and ST2/IL33R indicates that the mechanism of radiation-induced cardiotoxicity does not involve the atrial stress and stretch.

The monitoring of NLR, PLR, NT-proBNP, cTn I, and ST2/IL-33R in DTC/−T2DM patients receiving ^131^I therapy over a long period may help to identify those at risk of developing cardiac complications, including heart failure, myocardial complications, and chronic cardiomyopathies.

### 2.3. Correlations in the DTC/+T2DM Group

**Figure 4:**
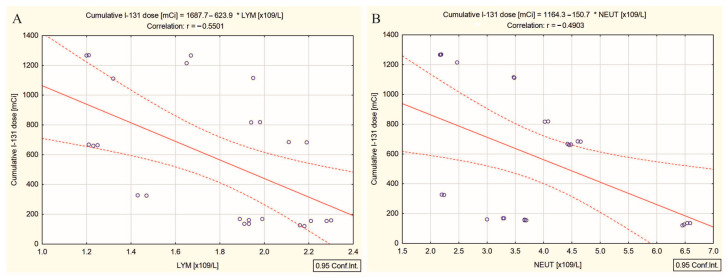
Correlations between (**A**) the cumulative doses of ^131^I and absolute lymphocyte count, and (**B**) the cumulative doses of ^131^I and absolute neutrophile count in differentiated thyroid cancer patients with type 2 diabetes mellitus; (“—” fitted linear regression curve, “- - -” equality line).

Our investigation into the DTC/+T2DM group yielded some intriguing results compared to the previous group of patients. No significant correlation was found between the cumulative doses of ^131^I and NLR, PLR, and the systemic immune-inflammation index (SII). The SII represents a novel composite index integrating two independent white blood cell subsets and platelets. However, the cumulative doses of ^131^I negatively correlated with the absolute lymphocyte count (r = −0.55, *p* = 0.004) (Figure 4A) and absolute neutrophil count (r = −0.49, *p* = 0.013) (Figure 4B).

**Figure 5:**
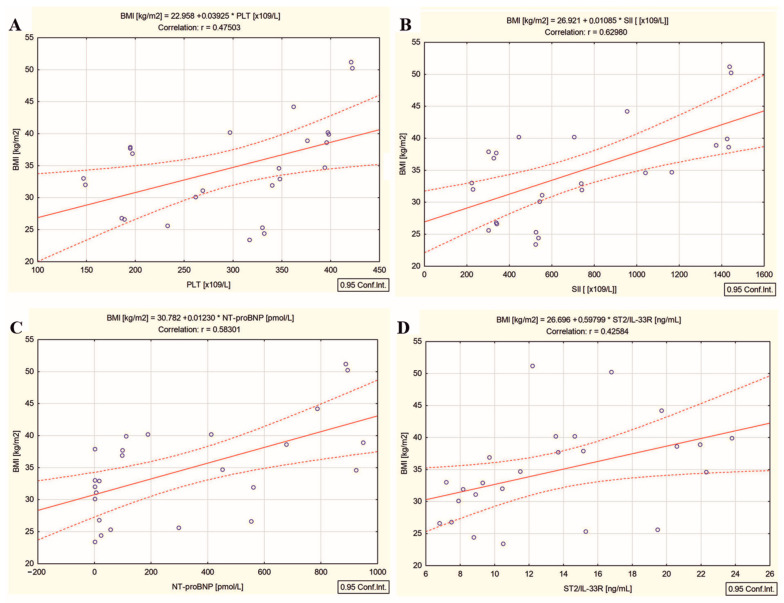
Correlations between (**A**) BMI and absolute platelet count, (**B**) BMI and SII, (**C**) BMI and NT-proBNP, and (**D**) BMI and ST2/IL-33R in differentiated thyroid cancer patients with type 2 diabetes mellitus; (“—” fitted linear regression curve, “- - -” equality line).

If, in the DTC/−T2DM overweight patients, BMI was not correlated with any analyzed blood parameter, in the DTC/+T2DM obese patients, BMI was correlated with absolute neutrophil count (r = 0.49, *p* = 0.012), absolute platelet count (r = 0.47, *p* = 0.016) (Figure 5A), NLR (r = 0.61, *p* = 0.001), PLR (r = 0.50, *p* = 0.01), SII (r = 0.62, *p* = 0.001) (Figure 5B), NT-proBNP (r = 0.58, *p* = 0.002) (Figure 5C) and ST2/IL-33R (r = 0.42, *p* = 0.034) (Figure 5D).

In T2DM, the heart is often under strain due to hyperglycemia, insulin resistance, autonomic dysfunction, and the increased risk of atherosclerosis [2]. In the context of T2DM, patients may already have an increased atrial volume or pressure load due to the increased blood volume (from fluid retention or poor kidney function), increased blood pressure (often associated with T2DM and comorbid conditions like hypertension) and diabetic cardiomyopathy, a condition where diabetes directly impacts the heart, causing systolic dysfunction, ventricular hypertrophy, and increased atrial pressure [2]. These underlying conditions increase the heart’s vulnerability to further damage when exposed to high doses of ^131^I.

**Figure 6:**
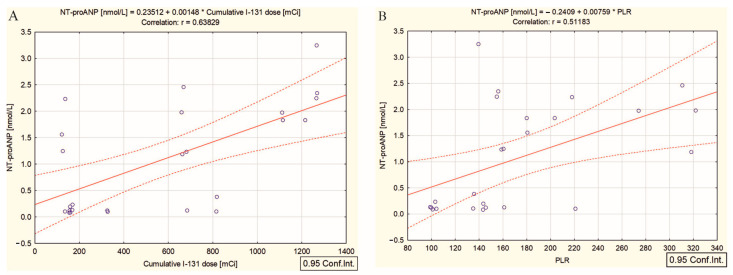
Correlation between (**A**) NT-proANP and the cumulative dose of ^131^I, and (**B**) NT-proANP and PLR in differentiated thyroid cancer patients with type 2 diabetes mellitus; (“—” fitted linear regression curve, “- - -” equality line).

Regarding the effect of high-dose irradiation, we found a positive correlation between the cumulative doses of ^131^I and NT-proANP (r = 0.63, *p* = 0.001) (Figure 6A) and no significant correlation with NT-proBNP, cTn I, and ST2/IL-33R. In response to the high cumulative doses of radiation, NT-proANP was correlated with the inflammatory biomarker PLR (r = 0.51, *p* = 0.009) (Figure 6B) and with NT-proBNP (r = 0.48, *p* = 0.015). Our results show that high cumulative doses of ^131^I may enhance pre-existing cardiovascular stress in DTC/+T2DM patients, leading to an increased pressure or volume overload in the atria, expressed by the higher NT-proANP serum concentrations than in the DTC/−T2DM group (*p* = 0.006). NT-proANP could signal atrial stretch and the early signs of atrial distress.

Given the presented results, NT-proANP could be used alongside NT-proBNP to assess the cardiotoxicity in patients with T2DM receiving ^131^I therapy. This could help clinicians identify patients at a high risk of atrial arrhythmias (atrial fibrillation) or early heart failure, enabling more targeted interventions to mitigate cardiac risk.

The presence of T2DM may exacerbate myocardial stress induced by high-dose irradiation, leading to essentially higher cTn I (as a subclinical myocardial injury biomarker) levels [9,11,21,22]. However, our results reveal a lower median cTn I serum concentration in DTC patients with T2DM than those without T2DM (*p* = 0.013) (Table 1), most likely as a long-lasting effect of metformin.

**Figure 7:**
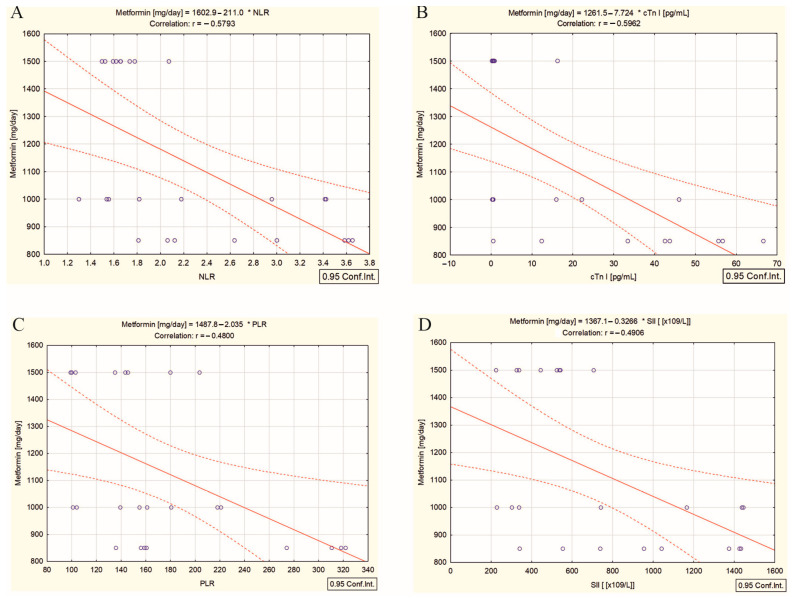
Correlation between (**A**) the metformin dose and NLR, (**B**) the metformin dose and cTn I, (**C**) the metformin dose and PLR, and (**D**) the metformin dose and SII in differentiated thyroid cancer patients with type 2 diabetes mellitus; (“—” fitted linear regression curve, “- - -” equality line).

It is well-known that cTn I is a specific biomarker of cardiac injury, especially myocardial necrosis [21,22,23]. Elevated serum concentrations are often used to diagnose acute myocardial infarction or cardiac injury [21,22,23]. After high cumulative doses of ^131^I, cTn I may increase due to necrosis or the injury to myocytes [24]. The radioprotective activity of metformin is highlighted by the dose-dependent increase in the lymphocyte count, although the correlation is not very strong (r = 0.40, *p* = 0.045). Regarding metformin’s cardioprotective effects, these are related to the reduction in subclinical inflammation and demonstrated by the inverse correlation with the folllowing: (i) inflammatory biomarkers NLR (r = −0.57, *p* = 0.002) (Figure 7A) and PLR (r = −0.48, *p* = 0.015) (Figure 7C); (ii) the systemic immune-inflammation index, SII (r = −0.49, *p* = 0.013) (Figure 7D) and (iii) the subclinical myocardial injury biomarker, cTn I (r = −0.59, *p* = 0.002) (Figure 7B). These findings suggest that metformin could attenuate the extent of cardiac injury, protecting cardiomyocytes from radiation-induced damage in patients with T2DM undergoing ^131^I therapy.

## 3. Discussion

The main findings of the present study regarding the CVD mechanisms behind ^131^I therapy in high doses include the following: (i) In DTC/−T2DM patients, high cumulative doses of ^131^I promote a pro-inflammatory state that accelerates the development of cardiotoxicity. Monitoring NLR, PLR, NT-proBNP, ST2/IL-33R, and cTn I in these patients may help identify those at risk of developing cardiac complications. (ii) In patients with T2DM and obesity, high-cumulative ^131^I doses lead to the release of NT-proANP, which signals that the atria are under significant stress. Increased NT-proANP levels might indicate the early stages of atrial remodeling, and NT-proANP could serve as a biomarker for early cardiac dysfunction in this population. (iii) In patients with T2DM receiving a three-fold higher dose of ^131^I than in patients without T2DM, metformin improves glucose control and offers cardiac protection by reducing radiation-induced cardiotoxicity. Its potential to lower cTn I levels in the face of ^131^I exposure could make it a valuable adjunctive therapy in these patients.

Several studies [14,15,16,17,18,19] have provided robust evidence that metformin, a biguanide drug that lowers glucose production by the liver, plays a significant role in cancer therapy. These studies have shown that metformin produces significant radiosensitization over time, attributed to various mechanisms, such as the re-oxygenation of the existing hypoxic tumor [15], activation of the adenosine-monophosphate-activated protein kinase (AMPK)/mammalian target of rapamycin (mTOR) pathway [16] and other microenvironmental considerations that are operative for the enhancement, particularly glucose concentration at the time of metformin administration [17]. The sodium iodide symporter (NIS) plays a pivotal role in ^131^I therapy, ensuring the effectiveness of the treatment by facilitating enough ^131^I uptake by the remaining thyroid tissue. Metformin exerts its effects through AMPK (a serine/threonine protein kinase) activation, leading to decreased NIS and thyroid iodine uptake in vitro and animal models [13,29]. Sloot et al. [29] have demonstrated that metformin use and a hypocaloric diet lead to AMPK activation in healthy volunteers without significant effects on thyroid iodine uptake. On the other hand, in a review, García-Sáenz et al. [30] noted that metformin could increase thyroid cancer cells’ sensitivity to radiation due to cell redifferentiation, with a subsequent increase in ^131^I uptake. Our results show that the ^131^I cumulative dose was higher in DTC/+T2DM patients treated with metformin than in the DTC/−T2DM group (*p* < 0.001). In our previous research [31], we showed that the whole-blood radioactivity measured three days after the ^131^I intake was significantly lower in the DTC/+T2DM patients than in the DTC/−T2DM patients (*p* < 0.001) despite higher administered doses. The explanations for this difference (higher ^131^I cumulative dose administered in DTC/+T2DM patients than in those with DTC/−T2DM) converge towards NIS from two directions: (i) the presence of T2DM: there is the possibility that ^131^I uptake may be sufficiently high in the pancreatic tissues (especially in the islets of Langerhans), which are known to exhibit dysfunction in T2DM patients, to reduce ^131^I uptake in the remnant thyroid tissue [32]; and (ii) metformin treatment: metformin activates AMPK, leading to decreased NIS and thyroid ^131^I uptake.

The number of ^131^I treatment cycles or high cumulative ^131^I doses were associated with a significant decrease in lymphocytes and platelet counts when Rui et al. [33] compared pre-^131^I with 4–6 months post-^131^I therapy. In our study, radiation-induced lymphopenia and thrombocytopenia were noticed only in the DTC/−T2DM group with significant positive correlations between cumulative doses of ^131^I and NLR, and PLR (r = 0.57 and r = 0.46, *p* < 0.001), but not with SII. Moreover, the similar negative correlation measured between the cumulative ^131^I dose and the absolute number of lymphocytes in the two groups (DTC/−T2DM: r = −0.58, *p* < 0.001 and DTC/+T2DM: r = −0.55, *p* = 0.004) confirms the fact that lymphocytes are the most radiosensitive cells in the human body [34].

However, despite the three times higher cumulative dose administered [660 mCi (24.42 GBq) vs. 209 mCi (7.73 GBq), *p* < 0.001], the absolute number of lymphocytes and platelets was higher in the patients with concurrent T2DM treated with metformin than in the patients without T2DM, and, of course, without metformin treatment (1.9 × 10^9^/L vs 1.4 × 10^9^/L, *p* = 0.015 and 330 × 10^9^/L vs 230 × 10^9^/L, *p* = 0.002). On the other hand, as we have previously shown, lymphocytes are the most sensitive to ^131^I therapy, regardless of the presence or absence of T2DM. In this interplay between ^131^I and metformin therapy, lymphocytes are in the middle. Our results align with those of authors who showed that metformin’s radioprotective activity protects lymphocytes from radiation damage [35,36]. The positive correlation between the metformin dose and the lymphocyte count (r = 0.40, *p* = 0.045) demonstrates metformin’s radioprotective properties, even though the relationship is not very strong. These findings, which align with Bikas et al.’s work [36], are significant as they demonstrate metformin’s dose-dependent radioprotective effects and its potential to accelerate blood count recovery post-radiotherapy.

Regarding the higher absolute number of platelets in DTC/+T2DM patients than in the DTC/−T2DM group (*p* = 0.002), we previously showed that targeted ^131^I therapy could be considered an external stimulus that could significantly activate platelets through platelet–neutrophil complexes [37]. This conclusion is supported by the negative correlation between the cumulative dose of ^131^I and the absolute number of neutrophils, as shown in Figure 4B, and the lack of correlation with the absolute platelet count. This relationship suggests that ^131^I triggers the early infiltration of neutrophils. Our results align with Raymakers et al. [38], who showed that neutrophils are among the first immune cells to infiltrate tumors after radiotherapy, demonstrating that they are essential for the initial antitumor immune response. On the other hand, Wisdom et al. [39] highlighted the role of neutrophils in promoting resistance to radiotherapy. The three-fold higher cumulative dose administered to DTC/+T2DM patients than those with DTC/−T2DM could be due, in addition, to metformin’s effect on decreasing NIS and thyroid ^131^I uptake, and to complex mechanisms, including neutrophil-mediated radiotherapy resistance processes. Further, given that T2DM is associated with a pro-thrombotic state due to the mutual activation of neutrophils and platelets, the increase in platelet count may be attributed to neutrophils both through direct interactions (via surface receptors) and indirect signaling (though the release of inflammatory mediators) in response to ^131^I. Platelet–neutrophil complexes further lead to platelet activation, and the inflammatory mediators, released by them, recruiting more platelets to the site of inflammation in response to ^131^I.

The results presented above confirm the hypothesis that metformin could act as a radioprotector in patients with T2DM who are exposed to high cumulative doses of ^131^I. Even though the radiation doses administered were three-fold higher than in patients without T2DM, metformin showed potential radioprotective effects, demonstrated by the reduction in inflammation caused by this high cumulative dose radiation exposure.

Female patients with DTC associated with T2DM are obese, with a median BMI of 34.6 kg/m^2^ (obesity class I). BMI was positively correlated with neutrophils, platelets (Figure 5A), NLR, PLR, and SII (Figure 5B) only in DTC/+T2DM patients, confirming the clinical picture of chronic low-grade systemic inflammation specific to T2DM [5]. Moreover, the statistically significant correlation between high BMI and high platelets (Figure 5A), high SII (Figure 5B), high NT-proBNP (Figure 5C), and high ST2/IL-33 (Figure 5D) marks a cross-link between obesity, thrombosis, systemic inflammation, myocardial inflammation, and myocardial wall stress unrelated to ^131^I therapy. These connections underscore the association between obesity and heightened platelet activation in the DTC/+T2DM group, establishing obesity as a significant risk factor for CVD disease due to its pro-thrombotic clinical condition without any connection to the action of ionizing radiation.

Moreover, among a panel of biomarkers, including NT-proBNP, ST2/IL-33R, cTn I, and NT-proANP, correlations were observed with the cumulative dose of ^131^I, but with differences between groups. If in the DTC/−T2DM group, the cumulative dose of ^131^I was correlated with NT-proBNP (Figure 1A), cTn I (Figure 1B), and ST2/IL-33R (Figure 1C), in the group of patients with T2DM, the radioactive dose was correlated only with NT-proANP (Figure 6A). The mentioned correlations demonstrate that the mechanisms of generating cardiotoxicity in the two groups of patients are different.

Targeted therapy with cumulative high doses of ^131^I can deplete parenchymal and vascular endothelial cells, with both macro- and microvascular effects, by two pathways involving cytotoxicity and inflammation [9,40]. In the case of patients with DTC/−T2DM, the activation of the inflammatory cascade is demonstrated by the significant positive correlations between the high cumulative dose of ^131^I and NLR and PLR (r = 0.57 and r = 0.46, *p* < 0.001). This cascade initially leads to cellular stress, which can significantly impact vascular permeability, promoting the degeneration of cells in myocardial capillaries [41] and subsequently, myocardial hypertrophy and fibrosis (significant positive correlation with ST2/IL-33R) (Figure 1C) [27], followed by an increase in myocardial wall stress and stretch (significant positive correlation with NT-proBNP) (Figure 1A) [21], and finally, cardiomyocyte necrosis and damage (significant positive correlation with cTn I) (Figure 1B) [19]. Our reasoning is based on the following findings: (i) positive correlations between the high dose radiation-induced inflammatory responses biomarkers NLR and PLR and cardiac biomarkers NT-proBNP (Figure 2A,D), cTn I (Figure 2B,E), and ST2/IL-33R (Figure 2C,F); and (ii) interconnecting relationships between all three cardiac biomarkers previously mentioned (NT-proBNP, cTn I, ST2/IL-33R), in response to radiation injury, as illustrated in Figure 3A–C.

The biomarker cascade suggests that high radiation promotes a pro-inflammatory state that accelerates the development of cardiotoxicity. The summary of the high cumulative ^131^I dose-induced cardiotoxicity mechanism in DTC/−T2DM patients would be as follows: (i) high-cumulative ^131^I doses trigger acute inflammation; (ii) inflammation leads to vascular permeability changes, which can cause fluid accumulation, myocardial degeneration, and capillary cell damage; (iii) the ST2-IL33 pathway is activated, leading to cardiac fibrosis, myocardial hypertrophy and stiffening and impaired heart function; (iv) increased wall stress and ventricular stretch, reflecting ventricular overload; and (v) cardiomyocyte necrosis and damage.

The mechanisms seem more complicated in patients with DTC/+T2DM because the circulating levels of NT-proBNP, NT-proANP, and ST2/IL-33R were higher, while the levels of cTn I were lower than in patients without T2DM. As we specified, this profile is related to T2DM and obesity, not the high ^131^I cumulative dose. That is why we hypothesize that metformin treatment could be involved. The results regarding the effect of metformin on NT-proBNP and cTn I are controversial. Alkuraishy et al. [42] showed that metformin reduced the serum cTn I level in patients with myocardial infarction, compared to control subjects. In contrast, a post hoc analysis of a 4.3-year randomized controlled trial found that metformin did not have a clinically significant effect on cTn I when compared to the placebo [43]. Unfortunately, we did not find any studies to follow the cardioprotective effect of metformin during ^131^I therapy. In our study, the lower median cTn I (as a subclinical myocardial injury biomarker) serum concentration in patients with T2DM, than those without T2DM (*p* = 0.013), could be a long-lasting effect of metformin, which has CVD benefits beyond its antihyperglycemic effects [18]. Several studies have shown that metformin influences the pathways involved in the complex interaction between immunity, inflammation, and metabolism. Zou et al. [44] demonstrated metformin’s antioxidant and anti-inflammatory actions through the downregulation of reactive oxygen species, leading to a subsequent reduction in neutrophil recruitment in zebrafish models of inflammation. NLR has recently been recognized as a predictor of poor outcomes in CVD patients (each one-unit increase in NLR is associated with a 15% higher risk of all-cause mortality and a 14% higher risk of cardiovascular mortality) [45]. Cameron et al. [46], in investigating the GoDARTS (Genetics of Diabetes Audit and Research in Tayside Scotland) patient database, found evidence of metformin reducing subclinical inflammation, as measured by NLR in these patients. Adel Mohammed et al. [47] showed that metformin therapy in patients with T2DM leads to a dose-dependent reduction in NLR. Our results align with these findings. The inverse correlations between metformin dose and NLR (Figure 7B), PLR (Figure 7C), SII (Figure 7D), and cTn I (Figure 7B) confirm metformin’s cadioprotective effects by suppressing inflammation in the DTC/+T2DM group.

Our findings showed that metformin, a common antidiabetic agent, has potential radioprotective and cardioprotective effects. It suppresses the inflammation caused by high-dose radiation exposure, leading to a dose-dependent reduction in cTn I in DTC/+T2DM patients. Metformin reduces cTnI levels by protecting cardiomyocytes from radiation-induced damage, thereby minimizing cardiac injury in patients receiving ^131^I therapy. Monitoring cTn I and considering the use of metformin as part of the therapeutic strategy could help manage cardiotoxicity in T2DM patients undergoing ^131^I therapy.

In another order of ideas, in the DTC/+T2DM group, the cumulative ^131^I dose correlated only with NT-proANP (Figure 6A). The diabetic and obese microenvironment can explain this different behavior because T2DM patients often have hypertension and fluid retention due to insulin resistance and metabolic disturbances [48]. NT-proANP is a sensitive marker of hemodynamic status that is less studied but recommended for obese patients’ cardiac evaluation [49]. It is well established that NT-proANP is a type of natriuretic peptide that promotes sodium and water excretion at the renal level, reducing blood volume and pressure [22,48]. Moreover, NT-proANP is characterized by its role in limited platelet activation through the cyclic GMP pathway, exhibiting anti-inflammatory effects (inhibits pro-inflammatory cytokines and reduces the infiltration of immune cells in tissues) and anti-fibrotic properties (protects the heart and vascular system from damage) [50]. As seen in a healthy system, NT-proANP exerts a protective mechanism. However, in T2DM patients undergoing ^131^I therapy, the mechanisms must be discussed in terms of the inflammatory response and cardiovascular stress induced by radiation injury. Sujana et al. [51], analyzing 11.537 subjects over 13.8 years of median follow-up, demonstrated that mid-regional proANP was inversely associated with incident T2DM. The positive relationship between the cumulative dose of ^131^I and NT-proANP serum concentration in the DTC/+T2DM group indicates that ^131^I therapy may amplify existing cardiovascular stress due to radiation-induced inflammation, thyroid hormone fluctuations, and altered fluid balance, stimulating the release of NT-proANP as a compensatory mechanism to alleviate these stresses (Figure 6A). Further, the extent to which NT-proANP can counteract platelet activation and inflammatory effects in these patients may be limited due to its impaired regulation, demonstrated by the lack of correlation between NT-proANP and the absolute platelet count and the significant statistical relationship between NT-proANP and PLR (Figure 6B). Thus, in the DTC/+T2DM group, ^131^I in high doses contributes, in time, to an increased burden on the heart’s atrial chambers. Over an extended period, the prolonged atrial hemodynamic overload may lead to persistent hemodynamic challenges, imposing stress on the myocardial walls. Our hypothesis is strengthened by the fact that in the DTC/+T2DM group, no interconnecting relationships exist between NT-proANP and the other cardiac biomarkers, except NT-proBNP (r = 0.48, *p* = 0.015), in response to radiation injury.

Our results show that the summary of the high cumulative ^131^I dose-induced cardiotoxicity mechanism in DTC/+T2DM patients would be as follows: (i) High-cumulative ^131^I doses might exacerbate pre-existing cardiovascular stress (due to hypertension, diabetic cardiomyopathy, or fluid retention) in T2DM patients, leading to increased pressure or volume overload in the atria, either due to direct radiation effects on the myocardium or secondary effects from systemic inflammation and fluid shifts. (ii) Atrial stretch (as a result of volume or pressure overload) leads to the release of NT-proANP, which signals that the atria are under significant stress. The increased NT-proANP circulating levels are part of the body’s attempt to relieve the overload by enhancing natriuresis and diuresis (excretion of sodium and water). (iii) Elevated NT-proANP levels might indicate the early stages of atrial remodeling and NT-proANP could serve as a biomarker for early cardiac dysfunction in this population. NT-proANP could be used alongside other biomarkers, like NT-proBNP and cTn I, to assess cardiotoxicity in patients with T2DM receiving ^131^I therapy. This would help clinicians identify T2DM patients who are at a high risk for atrial arrhythmias (such as atrial fibrillation) or heart failure early, enabling more targeted interventions to mitigate cardiac risks. The adjustment of treatment strategies, such as blood pressure management, the optimization of fluid balance, or the use of diuretics to manage volume overload, should be considered in T2DM patients with increased NT-proANP circulating levels. In addition, the close monitoring of arrhythmias and atrial fibrillation could help prevent further complications.

Several limitations of the current study should be considered. First, the sample size is relatively small. Despite this limitation, the study included patients referred from all over the country to our institute. In addition, the sample size, although small, was enough for statistical calculations with a 95% confidence interval. In addition, the sample size reflects that the association between DTC and T2DM is rare. On the other hand, the study population was composed of only women, due to sex-related differences in clinical outcomes and the interpretation of results, since sex hormones significantly influence the regulation of cardiovascular and immune functions. A complex crosstalk exists between estradiol, progesterone, androgens, thyroid hormones, cardiac biomarkers, and the immune system [52]. Future studies with larger, multicentric, more diverse cohorts (including males and females) will be necessary to assess the impact of ^131^I therapy and metformin use across different demographics. Another limitation lies in the fact that the present results were obtained from a monitoring period of only 53 months. More extended follow-up periods, ideally spanning more than 5 years, are essential for capturing delayed radiotoxicity and cardiotoxicity. Potential confounding factors, such as renal function, baseline cardiovascular health, and concurrent medications, may have influenced the outcomes, and future studies should control for these variables to isolate the effects of ^131^I therapy and metformin.

## 4. Materials and Methods

### 4.1. Patients and Study Protocol

This study involved 74 female patients with DTC/−T2DM (mean age, 58.1 ± 8.6 years) and 25 female patients with DTC/+T2DM (mean age, 61.4 ± 7.2 years), who were referred to the Department of Radionuclide Therapy of the Institute of Oncology Bucharest for ^131^I therapy over a period of approximately 53 months, spanning from 2015 to 2020. The patients received a cumulative oral dose of ^131^I sodium iodide ThyroTop exceeding 150 mCi (5.55 GBq). ThyroTop131, the radiopharmaceutical used, was sourced from the Institute of Isotopes Co., Ltd. (IZOTOP) in Budapest, Hungary. The inclusion criteria for patients in the study were clearly defined as follows: (i) age between 40 and 70 years old; (ii) non-smokers; (iii) the availability of the patient’s medical and drug history; (iv) the absence of a history of cardiovascular disease, including heart failure, acute coronary syndrome, symptomatic valvular dysfunction, or cardiomyopathy; (v) the absence of any signs of infection, bone marrow disorders, immune deficiency; (vi) the absence of poorly controlled diabetes; and (vii) the absence of poorly controlled hypertension. Only women were enrolled in the study to avoid intersex variations.

Demographic information, such as age, smoking status, BMI, cumulative ^131^I dosage/patient, daily dose of levothyroxine/patient, and daily dose of metformin/patient, were collected from medical records. The time assessment occurred during the most recent follow-up visit, approximately six months after the most recent therapeutic dose of ^131^I.

The study adhered to the ethical principles outlined in the Declaration of Helsinki and received approval from the Institute of Oncology Bucharest ethics committee (Approval No. 15140/9 October 2019). Furthermore, informed consent was obtained from all patients before participating in the study.

### 4.2. BMI Calculation

BMI was calculated as BMI = kg/m^2^, using the patient’s height and weight. The range for normal weight falls between 18.5 and 24.9 kg/m^2^. A BMI of 25 to 29.9 kg/m^2^ indicates overweight, while a BMI of 30 kg/m^2^ or higher signifies obesity. Obesity, according to BMI, is categorized as follows: class I (moderate obesity: 30 kg/m^2^ ≤ BMI < 35 kg/m^2^), class II (severe obesity: 35 kg/m^2^ ≤ BMI < 39.9 kg/m^2^), and class III (morbid obesity: 40 kg/m^2^ ≤ BMI) [28].

### 4.3. NLR, PLR and SII Calculation

An analysis of blood-count parameters (neutrophils, lymphocytes, and platelets) was performed using an ADVIA 2120i automatic Hematology System (Siemens Healthineers, Munich, Germany) with auto slide by Siemens on 2 mL of peripheral fasting blood collected into BD Vacutainer EDTA tubes from the cubital vein.

NLR and PLR were calculated as cumulative high-dose radiation-induced inflammatory response biomarkers by dividing the absolute neutrophil or platelet count by the lymphocyte count. The systemic immune-inflammation index, SII, is a powerful tool that reflects the interaction of thrombocytosis, inflammation, and immunity after a cumulative high dose of ^131^I. This index integrated three types of immune cells (platelets, neutrophils, and lymphocytes) and was calculated by the following formula [53]:

SII = platelet count [×10^9^/L] × neutrophil count [×10^9^/L]/lymphocyte count [10^9^/L] (1)

### 4.4. Biomarker Measurements

Venous blood specimens were collected from all patients on the day of their control visit. After collection, the blood samples were centrifuged at 2000× *g* at 4 °C. The serum samples were then aliquoted into labeled cryo-vials and frozen at −80 °C. The samples were frozen for a variable period of up to 12 months.

The biologically inactive molecules NT-proBNP (1–76) and NT-proANP (1–98) were selected for the study, over the biologically active peptides BNP and ANP, because they are more stable and have longer half-lives (120 min vs. 20 min and 60–120 min vs. 2.5 min, respectively) [54].

The quantitative determination of NT-proBNP (1–76), NT-proANP (1–98), cTn I, ST2/IL-33R was performed using commercially validated quantitative enzyme-linked immunosorbent assay (ELISA) kits (NT-proBNP and NT-proANP from Biomedica Medizinprodukte Gmbh, Vienna, Austria; cTn I from Abcam, Cambridge, UK and Human ST2/IL-33R Quantikine from R&D Systems, Inc., Minneapolis, MN, USA). Each kit followed the manufacturer’s instructions, which included pre-validated reagents, standards, and controls. The concentrations of NT-proBNP (1–76), NT-proANP (1–98), cTn I, and ST2/IL-33R were measured using standard sandwich ELISA, a sensitive and specific technique for detecting target proteins in serums. By employing commercially available ELISA kits with pre-validated reagents and a standardized protocol, reliable and reproducible measurements of NT-proBNP (1–76), NT-proANP (1–98), cTn I, and ST2/IL-33R were achieved. All assays were calibrated using a standard curve generated from serial dilutions of biomarker-specific standards included in the kits. The assay buffer was used as a blank to assess the background signal. The high and low controls were provided in the kits. NT-proBNP, NT-proANP, and cTn I biomarkers were assessed in duplicate in undiluted serum samples. The serum samples required a 20-fold dilution for ST2/IL-33R in a buffer protein base diluent. After that, ST2/IL-33R was assessed in duplicate in diluted serum samples. The absorbance values were plotted against standard concentrations to generate a standard curve using four-parameter logistic regression (4PL). The absorbance was read at 450 nm using a microplate reader (PR 1100 from Sanofi Pasteur, Bio-Rad Laboratories, Inc., Hercules, CA, USA), with a reference wavelength of 620 nm for background correction. The biomarker concentrations in the samples were interpolated from the curve.

According to the manufacturer, the values of the intra-assay precision were like those of the inter-assay precision, with the coefficients of variation ranging from 6.0 to 8.0% for NT-proBNP, 2.0 to 5.1% for NT-proANP, 3.0 to 7.1% for cTn I, and 4.4 to 5.6% for ST2/IL-33R. However, the precision (intra-assay variation) was tested with eight measurements of three different samples of known concentrations in one assay, and the reproducibility (inter-assay variation) for the same three samples was tested eight times in two assays. The intra- and inter-assay CVs were as follows: (i) 3.1% and 3.9%, respectively, at a mean concentration of 563.7 pmol/L for NT-proBNP; (ii) 2.3% and 3.2%, respectively, at a mean concentration of 1.07 nmol/L for NT-proANP; (iii) 4.5% and 5.6%, respectively, at a mean concentration of 10.27 ng/mL for ST2/IL-33R.

The storage at −80 °C for 12 months resulted in slight decreases (between 0.3% and 1.4%) in NT-proBNP, NT-proANP, cTn I, and ST2/IL-33R concentrations, compared to samples thawed after 24 h of storage.

### 4.5. Data Analysis

The study’s data analysis was carried out using several software packages, including Statistica 8.0 by StatSoft, Inc. (Tulsa, OK, USA), IBM SPSS Statistics Version 28.0.1.0, and Microsoft Office Excel 2007 SP2. Before the study, a power analysis was conducted to determine the necessary sample size for detecting statistically significant differences. The study was adequately powered (80% power) to detect meaningful relationships between the analyzed parameters. The alpha level (type I error rate) was set to 0.05 to ensure a 5% probability of incorrectly rejecting the null hypothesis. Descriptive statistics [mean, median, standard deviation, and interquartile range (IQR: 25–75%)] were calculated for continuous and categorical variables. The robustness of our analysis was ensured using the Shapiro–Wilk and Kolmogorov–Smirnov tests, commonly used to assess whether data follow a normal distribution. Regarding the inferential statistics, the non-parametric Kruskal–Wallis test was used to compare the distribution of continuous variables between different categories for independent samples (DTC/−T2DM group vs. DTC/+T2DM group). Pearson’s correlation coefficient (r) was used to explore the associations between the analyzed parameters. A multivariable analysis was used to evaluate the independent relationships between the analyzed parameters while adjusting for potential confounders that could influence the observed outcomes, like age or BMI. The study’s statistical significance was determined using a *p*-value threshold of less than 0.05.

## 5. Conclusions

In summary, the results of the present study suggest distinct high-cumulative ^131^I dosage-induced cardiotoxicity mechanisms in the two groups of patients.

In DTC/−T2DM patients, high cumulative doses of ^131^I promote a pro-inflammatory state that accelerates the development of cardiotoxicity. Monitoring NLR, PLR, NT-proBNP, ST2/IL-33R, and cTn I in these patients receiving ^131^I treatment may help identify those at risk of developing cardiac complications, including heart failure, myocardial fibrosis, and chronic cardiomyopathy. By understanding this high-cumulative ^131^I dosage-induced cardiotoxicity mechanism and its associated biomarkers, clinicians can better stratify risk, guide early interventions, and monitor therapeutic efficacy to mitigate long-term cardiovascular damage.

In patients with T2DM and obesity, high-cumulative ^131^I doses lead to the release of NT-proANP, which signals that the atria are under significant stress. Increased NT-proANP concentrations might indicate the early stages of atrial remodeling, and NT-proANP could serve as a biomarker for early cardiac dysfunction in this population. The measurement of circulating NT-proANP levels would help clinicians identify T2DM patients at a high risk for atrial arrhythmias (such as atrial fibrillation) or heart failure early, enabling more targeted interventions to mitigate cardiac risks. The adjustment of treatment strategies, such as blood pressure management, the optimization of fluid balance, or the use of diuretics to manage volume overload, should be considered in T2DM patients with increased NT-proANP circulating levels. In addition, the close monitoring of arrhythmias and atrial fibrillation could help prevent further complications.

Our results confirm the hypothesis that metformin could act as a cardioprotector in patients with T2DM exposed to high cumulative doses of ^131^I. Even though the radiation doses administered were three-fold higher than in patients without T2DM, metformin showed potential radioprotective and cardioprotective effects, demonstrated by the reduction in inflammation caused by this high cumulative dose radiation exposure. Its anti-inflammatory effects may help mitigate radiation-induced damage to the heart, leading to a dose-dependent reduction in cTn I in DTC/+T2DM patients. Metformin reduces cTn I level by protecting cardiomyocytes from radiation-induced damage, thereby minimizing cardiac injury in patients receiving ^131^I therapy. Monitoring cTn I and NT-proANP, and considering the use of metformin as part of the therapeutic strategy, could help manage cardiotoxicity in T2DM patients undergoing ^131^I therapy.

Understanding the differences in high-cumulative ^131^I dosage-induced cardiotoxicity mechanisms between the two groups is crucial for tailoring interventions and optimizing care for individuals undergoing ^131^I therapy for DTC, particularly considering their comorbidities, such as T2DM. The use of metformin as part of the therapeutic strategy could help manage cardiotoxicity in T2DM patients undergoing ^131^I therapy. Future research directions on oxidative stress, DNA damage, mitochondrial dysfunction, or inflammatory pathways driving ^131^I-induced radiotoxicity and cardiotoxicity are required to confirm these findings. The complex puzzle of the effect of high cumulative doses of ^131^I on the cardiovascular system in DTC with/without T2DM still needs to be fully solved.

## Figures and Tables

**Table 1:** Clinical, hematological and biochemical data in the study groups.

Variables	DTC/−T2DM	DTC/+T2DM	*p*-Value
	n = 74	n = 25	
Age (years) ^a^	58.1 ± 8.7	61.4 ± 7.2	0.147
BMI (kg/m^2^) ^b^	29.0 (26.2–33.2)	34.6 (30.1–38.9)	0.003
Cumulative ^131^I dose (mCi) ^b^	209.0 (151.2–582.0)	660.0 (158.0–818.0)	<0.001
Levothyroxine dose (mcg/day) ^b^	107.1 (85.2–148.6)	107.8 (87.9–149.8)	0.311
Metformin dose (mg/day) ^b^	-	1000 (850–1500)	-
Lymphocytes (×10^9^/L) ^b^	1.4 (1.1–1.8)	1.9 (1.4–2.1)	0.015
Neutrophils (×10^9^/L) ^b^	3.7 (3.1–4.4)	3.6 (3.0–4.5)	0.642
Platelets (×10^9^/L) ^b^	230.0 (193.0–283.0)	330.0 (197.0–376.0)	0.002
NLR ^b^	2.5 (1.8–3.4)	1.8 (1.6–2.9)	0.150
PLR ^b^	152.9 (119.7–200.0)	156.2 (135.8–203.6)	0.629
SII (×10^9^/L) ^b^	605.0 (338.4–844.9)	719.8 (386.5–1041.0)	0.142
NT-proBNP (pmol/L) ^b^	11.7 (1.9–168.5)	112.3 (16.7–561.2)	0.001
NT-proANP (nmol/L) ^b^	0.09 (0.08–0.13)	1.18 (0.12–1.97)	0.006
cTn I (pg/mL) ^b^	14.7 (5.3–35.9)	0.8 (0.5–33.5)	0.013
ST2/IL-33R (ng/mL) ^b^	9.8 (7.4–14.8)	12.2 (8.9–16.8)	0.033
TSH (IU/L) ^b^	0.27 (0.14–0.32)	0.39 (0.19–0.43)	0.070

BMI—body mass index; DTC/−T2DM—differentiated thyroid cancer without type 2 diabetes mellitus; DTC/+T2DM—differentiated thyroid cancer associated with type 2 diabetes mellitus; cTn I—cardiac troponin I; NT-proANP—N-terminal pro atrial natriuretic peptide; NT-proBNP—N-terminal pro brain natriuretic peptide; ST2/IL-33R—receptor of interleukin-33; NLR—neutrophil-to-lymphocyte ratio; PLR—platelet-to-lymphocyte ratio; ^131^I—radioiodine; SII—systemic immune-inflammation index; TSH—thyroid-stimulating hormone; ^a^ mean ± standard deviation; ^b^ Data are expressed as median and interquartile ranges (25–75%).

## Data Availability

All the data generated or analyzed during this study are included in the manuscript.

## References

[1] Magliano D.J., Boyko E.J. (2021). IDF Diabetes Atlas 10th Edition Scientific Committee.

[2] Galicia-Garcia U., Benito-Vicente A., Jebari S., Larrea-Sebal A., Siddiqi H., Uribe K.B., Ostolaza H., Martín C. (2020). Pathophysiology of Type 2 Diabetes Mellitus. Int. J. Mol. Sci..

[3] Ye J., Wu Y., Yang S., Zhu D., Chen F., Chen J., Ji X., Hou K. (2023). The global, regional and national burden of type 2 diabetes mellitus in the past, present and future: A systematic analysis of the Global Burden of Disease Study 2019. Front. Endocrinol..

[4] Seo Y.G., Choi H.C., An A.R., Park D.J., Park Y.J., Lee K.E., Park S.K., Hwang Y., Cho B. (2017). The Association between Type 2 Diabetes Mellitus and Thyroid Cancer. J. Diabetes Res..

[5] Brenta G., Di Fermo F. (2024). Thyroid cancer and insulin resistance. Rev. Endocr. Metab. Disord..

[6] Roh E., Noh E., Hwang S.Y., A Kim J., Song E., Park M., Choi K.M., Baik S.H., Cho G.J., Yoo H.J. (2021). Increased Risk of Type 2 Diabetes in Patients with Thyroid Cancer After Thyroidectomy: A Nationwide Cohort Study. J. Clin. Endocrinol. Metab..

[7] Fugazzola L., Elisei R., Fuhrer D., Jarzab B., Leboulleux S., Newbold K., Smit J. (2019). 2019 European Thyroid Association Guidelines for the Treatment and Follow-Up of Advanced Radioiodine-Refractory Thyroid Cancer. Eur. Thyroid J..

[8] Gherghe M., Lazar A.M., Mutuleanu M.D., Stanciu A.E., Martin S. (2022). Radiomics Analysis of [^18^F]FDG PET/CT Thyroid Incidentalomas: How Can It Improve Patients’ Clinical Management? A Systematic Review from the Literature. Diagnostics.

[9] Zhang H., Xie H., Li L. (2024). Association of radioactive iodine treatment in differentiated thyroid cancer and cardiovascular death: A large population-based study. J. Endocrinol. Investig..

[10] Mei X., Yao X., Feng F., Cheng W., Wang H. (2021). Risk and outcome of subsequent malignancies after radioactive iodine treatment in differentiated thyroid cancer patients. BMC Cancer.

[11] Tsai W.H., Zeng Y.H., Lee C.C., Chien M.N., Liu S.C., Chien K.L., Cheng S.P., Tseng P.J., Tsai M.C. (2023). Association between thyroid cancer and cardiovascular disease: A meta-analysis. Front. Cardiovasc. Med..

[12] Foretz M., Guigas B., Viollet B. (2023). Metformin: Update on mechanisms of action and repurposing potential. Nat. Rev. Endocrinol..

[13] Pappa T., Alevizaki M. (2013). Metformin and thyroid: An update. Eur. Thyroid J..

[14] Li H., Chen Y., Hu L., Yang W., Gao Z., Liu M., Tao H., Li J. (2023). Will metformin use lead to a decreased risk of thyroid cancer? A systematic review and meta-analyses. Eur. J. Med. Res..

[15] Rattan R., Ali Fehmi R., Munkarah A. (2012). Metformin: An emerging new therapeutic option for targeting cancer stem cells and metastasis. J. Oncol..

[16] Chomanicova N., Gazova A., Adamickova A., Valaskova S., Kyselovic J. (2021). The role of AMPK/mTOR signaling pathway in anticancer activity of metformin. Physiol. Res..

[17] Brown S.L., Kolozsvary A., Isrow D.M., Al Feghali K., Lapanowski K., Jenrow K.A., Kim J.H. (2019). A Novel Mechanism of High Dose Radiation Sensitization by Metformin. Front. Oncol..

[18] Yu J.M., Hsieh M.C., Qin L., Zhang J., Wu S.Y. (2019). Metformin reduces radiation-induced cardiac toxicity risk in patients having breast cancer. Am. J. Cancer Res..

[19] Siteni S., Barron S., Luitel K., Shay J.W. (2024). Radioprotective effect of the anti-diabetic drug metformin. PLoS ONE.

[20] Pacini F., Fuhrer D., Elisei R., Handkiewicz-Junak D., Leboulleux S., Luster M., Schlumberger M., Smit J.W. (2022). 2022 ETA Consensus Statement: What are the indications for post-surgical radioiodine therapy in differentiated thyroid cancer?. Eur. Thyroid J..

[21] Lyon A.R., López-Fernández T., Couch L.S., Asteggiano R., Aznar M.C., Bergler-Klein J., Boriani G., Cardinale D., Cordoba R., Cosyns B. (2022). ESC Scientific Document Group. 2022 ESC Guidelines on cardio-oncology developed in collaboration with the European Hematology Association (EHA), the European Society for Therapeutic Radiology and Oncology (ESTRO) and the International Cardio-Oncology Society (IC-OS). Eur. Heart J..

[22] Hinrichs L., Mrotzek S.M., Mincu R.I., Pohl J., Röll A., Michel L., Mahabadi A.A., Al-Rashid F., Totzeck M., Rassaf T. (2020). Troponins and Natriuretic Peptides in Cardio-Oncology Patients-Data from the ECoR Registry. Front. Pharmacol..

[23] Ulndreaj A., Brinc D., Altan M., Pons-Belda O.D., Fernandez-Uriarte A., Mu-Mosley H., Fattah F., von Itzstein M.S., Soosaipillai A., Kulasingam V. (2022). Quantitation of cardiac troponin I in cancer patients treated with immune checkpoint inhibitors: A case-control study. Clin. Chem. Lab. Med..

[24] Xiao H., Wang X., Li S., Liu Y., Cui Y., Deng X. (2021). Advances in Biomarkers for Detecting Early Cancer Treatment-Related Cardiac Dysfunction. Front. Cardiovasc. Med..

[25] Riccardi M., Myhre P.L., Zelniker T.A., Metra M., Januzzi J.L., Inciardi R.M. (2023). Soluble ST2 in Heart Failure: A Clinical Role beyond B-Type Natriuretic Peptide. J. Cardiovasc. Dev. Dis..

[26] Gherghe M., Lazar A.M., Mutuleanu M.-D., Bordea C.I., Ionescu S., Mihaila R.I., Petroiu C., Stanciu A.E. (2023). Evaluating Cardiotoxicity in Breast Cancer Patients Treated with HER2 Inhibitors: Could a Combination of Radionuclide Ventriculography and Cardiac Biomarkers Predict the Cardiac Impact?. Cancers.

[27] Yasen X., Aikebaier R., Maimaiti A., Mushajiang M. (2024). IL-33/soluble ST2 axis is associated with radiation-induced cardiac injury. Open Life Sci..

[28] Güneş S.B., Aytutuldu G.K., Akıncı B. (2024). Obesity awareness-insight is inversely associated with body composition and physical activity behaviour in women with obesity at the admission to a lifestyle modification program. J. Public Health.

[29] Sloot Y.J.E., Janssen M.J.R., van Herwaarden A.E., Peeters R.P., Netea-Maier R.T., Smit J.W.A. (2019). The Influence of Energy Depletion by Metformin or Hypocaloric Diet on Thyroid Iodine Uptake in Healthy Volunteers: A Randomized Trial. Sci. Rep..

[30] García-Sáenz M., Lobaton-Ginsberg M., Ferreira-Hermosillo A. (2022). Metformin in Differentiated Thyroid Cancer: Molecular Pathways and Its Clinical Implications. Biomolecules.

[31] Stanciu A.E., Hurduc A., Stanciu M.M., Gherghe M., Gheorghe D.C., Prunoiu V.M., Zamfir-Chiru-Anton A. (2023). Portrait of the Inflammatory Response to Radioiodine Therapy in Female Patients with Differentiated Thyroid Cancer with/without Type 2 Diabetes Mellitus. Cancers.

[32] Samadi R., Shafiei B., Azizi F., Ghasemi A. (2017). Radioactive Iodine Therapy and Glucose Tolerance. Cell J..

[33] Rui Z., Wu R., Zheng W., Wang X., Meng Z., Tan J. (2021). Effect of ¹³¹I Therapy on Complete Blood Count in Patients with Differentiated Thyroid Cancer. Med. Sci. Monit..

[34] Paganetti H. (2023). A review on lymphocyte radiosensitivity and its impact on radiotherapy. Front. Oncol..

[35] Sun X., Dong M., Gao Y., Wang Y., Du L., Liu Y., Wang Q., Ji K., He N., Wang J. (2022). Metformin increases the radiosensitivity of non-small cell lung cancer cells by destabilizing NRF2. Biochem. Pharmacol..

[36] Bikas A., Van Nostrand D., Jensen K., Desale S., Mete M., Patel A., Wartofsky L., Vasko V., Burman K.D. (2016). Metformin Attenuates 131I-Induced Decrease in Peripheral Blood Cells in Patients with Differentiated Thyroid Cancer. Thyroid.

[37] Stanciu A.E., Stanciu M.M., Zamfirescu A., Gheorghe D.C. (2022). Cardiovascular Effects of Cumulative Doses of Radioiodine in Differentiated Thyroid Cancer Patients with Type 2 Diabetes Mellitus. Cancers.

[38] Raymakers L., Demmers T.J., Meijer G.J., Molenaar I.Q., van Santvoort H.C., Intven M.P.W., Leusen J.H.W., Olofsen P.A., Daamen L.A. (2024). The Effect of Radiation Treatment of Solid Tumors on Neutrophil Infiltration and Function: A Systematic Review. Int. J. Radiat. Oncol. Biol. Phys..

[39] Wisdom A.J., Hong C.S., Lin A.J., Xiang Y., Cooper D.E., Zhang J., Xu E.S., Kuo H.-C., Mowery Y.M., Carpenter D.J. (2019). Neutrophils promote tumor resistance to radiation therapy. Proc. Natl. Acad. Sci. USA.

[40] Yang E.H., Marmagkiolis K., Balanescu D.V., Hakeem A., Donisan T., Finch W., Virmani R., Herrman J., Cilingiroglu M., Grines C.L. (2021). Radiation-Induced Vascular Disease-A State-of-the-Art Review. Front. Cardiovasc. Med..

[41] Ell P., Martin J.M., Cehic D.A., Ngo D.T.M., Sverdlov A.L. (2021). Cardiotoxicity of Radiation Therapy: Mechanisms, Management, and Mitigation. Curr. Treat. Options Oncol..

[42] Alkuraishy H.M., Al-Gareeb A.I. (2015). New Insights into the Role of Metformin Effects on Serum Omentin-1 Levels in Acute Myocardial Infarction: Cross-Sectional Study. Emerg. Med. Int..

[43] Stultiens J.M.G., Top W.M.C., Kimenai D.M., Lehert P., Bekers O., Stehouwer C.D.A., Kooy A., Meex S.J.R. (2022). Metformin and high-sensitivity cardiac troponin I and T trajectories in type 2 diabetes patients: A post-hoc analysis of a randomized controlled trial. Cardiovasc. Diabetol..

[44] Zhou R., Ding R.-C., Yu Q., Qiu C.-Z., Zhang H.-Y., Yin Z.-J., Ren D.-L. (2024). Metformin Attenuates Neutrophil Recruitment through the H3K18 Lactylation/Reactive Oxygen Species Pathway in Zebrafish. Antioxidants.

[45] Li X., Liu M., Wang G. (2024). The neutrophil–lymphocyte ratio is associated with all-cause and cardiovascular mortality in cardiovascular patients. Sci. Rep..

[46] Cameron A.R., Morrison V.L., Levin D., Mohan M., Forteath C., Beall C., McNeilly A.D., Balfour D.J., Savinko T., Wong A.K. (2016). Anti-Inflammatory Effects of Metformin Irrespective of Diabetes Status. Circ. Res..

[47] Adel Mohammed M., Hussein N.R., Al-Niemi M.S., Alkuraishy H.M. (2021). The Potential Effect of Metformin Therapy on Neutrophil-Lymphocyte Ratio in Patients with Type II Diabetes Mellitus: A New Horizon. Int. J. Pharm. Res..

[48] Lu X., Xie Q., Pan X., Zhang R., Zhang X., Peng G., Zhang Y., Shen S., Tong N. (2024). Type 2 diabetes mellitus in adults: Pathogenesis, prevention and therapy. Signal Transduct. Target. Ther..

[49] Sundar Singh S.D., Pithadia A.B., Lavanya S. (2022). A Review on Assessment of Cardiovascular Risk in Type II Diabetes Patients by Using Mr-proANP and NTproBNP. Clin. Case Rep. Int..

[50] Della Corte V., Pacinella G., Todaro F., Pecoraro R., Tuttolomondo A. (2023). The Natriuretic Peptide System: A Single Entity, Pleiotropic Effects. Int. J. Mol. Sci..

[51] Sujana C., Salomaa V., Kee F., Costanzo S., Söderberg S., Jordan J., Jousilahti P., Neville C., Iacoviello L., Oskarsson V. (2021). Natriuretic Peptides and Risk of Type 2 Diabetes: Results from the Biomarkers for Cardiovascular Risk Assessment in Europe (BiomarCaRE) Consortium. Diabetes Care.

[52] Ren B., Zhu Y. (2022). A New Perspective on Thyroid Hormones: Crosstalk with Reproductive Hormones in Females. Int. J. Mol. Sci..

[53] Xia Y., Xia C., Wu L., Li Z., Li H., Zhang J. (2023). Systemic Immune Inflammation Index (SII), System Inflammation Response Index (SIRI) and Risk of All-Cause Mortality and Cardiovascular Mortality: A 20-Year Follow-Up Cohort Study of 42,875 US Adults. J. Clin. Med..

[54] Çelik A., Kılıçaslan B., Temizhan A., Güvenç T.S., Altay H., Çavuşoğlu Y., Yılmaz M.B., Yıldırımtürk Ö., Nalbantgil S., Ural D. (2023). How to Use Natriuretic Peptides in Patients with Heart Failure with Non-Reduced Ejection Fraction?. Anatol. J. Cardiol..

